# Psychometric Properties of the Persian Version of the Difficulties in Emotion Regulation Scale) DERS-6 & DERS-5- Revised (in an Iranian Clinical Sample 

**Published:** 2015-04

**Authors:** Mina Mazaheri

**Affiliations:** 1 Psychosomatic Research Center, Isfahan University of Medical Sciences, Isfahan, Iran

**Keywords:** *Persian Version of the Difficulties in Emotion Regulation Scale (Scales of 6 &amp; 5-factors)*, *Clinical Sample*, *Reliability and Validity*

## Abstract

**Objective:** The purpose of this study was to determine the construct validity and reliability of the two forms of the Persian version of the Difficulties in Emotion Regulation Scale (DERS-6 & DERS-5-revised) in a clinical sample.

**Methods**: The clinical sample consisted of 181 patients diagnosed with Functional GI Disorders (FGID) who referred to the digestive psychosomatic clinic in Isfahan in 2012. They were selected by census method (In a given period of time). The Persian version of the DERS, the short form of the DASS, and the TAS-20 were used to collect data.

**Results:** The results of the factor structure or construct validity using principal components analysis with varimax rotation recognized 7 factors for the DERS-6 (Goals, Awarness, Impalse, Non Acceptance, Strategy, Clarity, Recognition), and 6 factors for the DERS-5- revised (Non Acceptance, Goals, Impalse, Strategy, Clarity, Recognition) in the clinical sample. They showed the common variance of 59.51% and 59.15%, respectively. Also, the results showed that the concurrent validity of both forms of the DERS and most of their factors, and their reliability in terms of Cronbach-Alpha were favorable.

**Discussion:** Considering the factor structure and favorable psychometric properties of the two scales of DERS-6 & DERS-5-revised, the scales can be used in clinical samples.

One of the important psychological aspects of human is emotions. Emotional experiences have an important role in everyday life, psychological health, motivational processes, social vicissitude and the appropriate response to stressful events, and can affect different actions of individuals (1). However, emotions are not always helpful; they can hurt as well as help us (2). They do so when they are of the wrong type, when they come at the wrong time, or when they occur at the wrong intensity level (3). Since individuals are able to influence the intensity, duration and type of their emotional experiences (1), the necessity of emotion regulation is important. Emotion regulation shows that how we influence our emotions, and how we experience and express these emotions (4) 

The disability or difficulty to experience and differentiate emotions may be as maladaptive as defects in the ability to modulate strong negative emotions (5, 6). Contemporary emotion theories emphasize the ways emotions facilitate adaptation 

(3). The researcher has suggested that adaptive emotion regulation involves changing the intensity or duration of an emotion rather than altering the experienced emotion (7, 8). In other words, adaptive regulation involves modulating the experience of emotions rather than eliminating certain emotions. It is thought that this modulation of arousal reduces the intensity of emotion so that the individual is able to control her or his behavior (9).

Based on the psychometric approach, emotion regulation is a multidimensional concept that is composed of the (a) awareness and understanding of emotions, (b) acceptance of emotions, (c) ability to control impulsive behaviors and behaving in accordance with desired goals when experiencing negative emotions, and (d) ability to use situationally appropriate emotion regulation strategies flexibly to modulate emotional responses in order to meet individual goals and situational demands. The relative absence of any or all these abilities indicates the presence of difficulties in emotion regulation or emotion dysregulation (9).

Deficits in emotion regulation appear to be related to development, maintenance and treatment of various forms of psychopathology. Evidence demonstrates that deficits in the ability to adaptively cope with challenging emotions are related to depression, borderline personality disorder, substance-use disorders, eating disorders, somatoform disorders and a variety of other psychopathological symptoms (10). According to Campbell-Sills, 2007, difficulties in emotion regulation form the underlying mechanisms of anxiety and mood disorders. Anxious and depressed individuals try to inhibit their negative emotions, and this causes the feelings to recur or intensify (11). 

Also, emotions seem to play an important role in initiating and sustaining psychosomatic complaints (12). The tendency and experimental instructions to inhibit emotional expression have been associated with increased physiological arousal (13, 14). Studies have reported that problematic emotions regulation such as anger and anxiety are involved in somatic problems like cardiovascular and gastrointestinal diseases (15, 16). Thus, emotions can have a substantial role in mental disorders. Indeed, more than half of the Axis I clinical disorders and all of the Axis II personality disorders involve problematic emotional responses (3). Considering the role of emotion regulation in the development and persistence of mental disorders, preparing an optimal tool to study the problems related to emotion regulation in research and clinical domains is of high importance. 

The Difficulties in Emotion Regulation Scale) DERS) was developed by Gratz & Roemer (2004) to assess emotion dysregulation more comprehensively than the existing measures (Negative Mood Regulation Scale & Meta-mood Trait Scale both emphasize the avoidance of negative emotions). The DERS can distinguish adaptive emotion regulation from emotional avoidance and expressive control. Factor structure and reliability of the DERS were studied in a normal sample. The results suggested the presence of six factors in emotion dysregulation: (1) Non-acceptance of emotional responses that reflects a tendency to have negative secondary emotional responses to one’s negative emotions, or nonaccepting reactions to one’s distress; (2) Difficulties engaging in goal-directed behavior that reflects difficulties concentrating and accomplishing tasks when experiencing negative emotions; (3) Impulse control difficulties that reflects difficulties remaining in control of one’s behavior when experiencing negative emotions; (4) Lack of emotional awareness that reflects the tendency to attend to and acknowledge emotions; (5) Limited access to emotion regulation strategies that reflects the belief that there is little that can be done to regulate emotions effectively once an individual is upset; (6) Lack of emotional clarity that reflects the extent to which individuals know (and are clear about) the emotions they are experiencing. To assess the reliability and determining the internal consistency of the DERS, Cronbach’s α was calculated. Results indicated that the DERS had high internal consistency (0.93); also, all the DERS factors had adequate internal consistency, with Cronbach’s α>:80 for each factor (9).

Most studies (17, 18, 19, 20, 21) have obtained a similar factor structure (6 factors) for the Difficulties in Emotion Regulation Scale developed by Graz and Roemer (2004) in normal adult subjects. The study of Tejeda et al. (2012) identified 4 factors in a sample of adolescents (22). By conducting a study on an Iranian normal sample, khanzadeh et al. (2012) revealed eight factors for this scale, but two factors were excluded due to loading only one item (11). The findings of various studies (18, 19, 20, 21, 22) have confirmed favorable reliability and concurrent validity of this scale. 

Gratz and Roemer (2004) designed the DERS to assess factors of the same underlying construct (i.e., emotion regulation). Therefore, the DERS factors should share significant intercorrelations and possess a consistent pattern of convergence with variables relevant to the emotion regulation domain. However, despite the fact that five of the DERS factors had moderate to strong intercorrelations (from 0.32 to 0.63), awareness factor demonstrated partly mild intercorrelations with the other factors (from .08 to 0.46) (23). Neumann et al. (2010) reached the almost similar conclusion (24). Tull et al. (2007) & (2010) found that the awareness factor was correlated only with the Clarity factor. Due to modest intercorrelations with the other DERS factors, awarness showed a relatively divergent pattern of relations with variables relevant to the extent of emotion regulation (25, 26). Bardeen et al. (2012) with examination of latent factor intercorrelations and a confirmatory factor analysis (CFA) suggested that the awarness factor may not represent the same emotion regulation construct as the other five DERS factors. Moreover, their findings supported the adequacy of a revised five-factor model of the DERS in which the awarness factor was removed. The DERS-5-revised model did not reduce concurrent relations between the DERS and variables relevant to the extent of emotion regulation (23).

To better assess the relationships between emotion regulation difficulties and phenomena relevant to this domain, it is necessary to have a valid scale to evaluate the domain of emotion regulation. According to the results of various studies, both DERS-6 & DERS-5-revised are instruments with favorable psychometric properties for research in the field of emotion regulation and problems relevant to it in the normal population. Because of the comprehensiveness of the scales for assessing the effective variables on emotion regulation, it seems necessary to evaluate factor structure and psychometric properties of the scales in the clinical groups with emotional disorders. Hence, the purpose of this study was to determine construct validity and reliability of both forms of the Persian version of the Difficulties in Emotion Regulation Scale (DERS-6 & DERS-5-revised) in a clinical sample.

## Material and Methods

*Participants*

 In this descriptive correlational study, 181(33 males and 148 females) patients with Functional GI Disorders (FGID), who referred to a digestive psychosomatic clinic in Isfahan in 2012, were selected by census sampling method (In almost 10 months). The mean age of the participants was 36.34 years and their age range was 18-66 years. The researcher ensured the patients that their information will remain confidential. 

*Measurements*

The Difficulties in Emotion Regulation Scale) DERS): The scale is a self-report measure developed to assess clinically relevant difficulties in emotion regulation. It has 36 items that are rated on a five-point Likert scale, ranging from 1 (almost never) to 5 (almost always). 11 (1, 2, 6, 7, 8, 10, 17, 20, 22, 24, 34) items are rated inversely. DERS items are recoded so that higher scores in every case indicate greater difficulties in emotion regulation (i.e., greater emotion dysregulation). The scale is composed of 6 factors: Non-acceptance of emotional responses (Non-Acceptance); difficulties engaging in goal-directed behavior (Goal); impulse control difficulties (Impulse); lack of emotional awareness (Awareness); limited access to emotion regulation strategies (Strategy); lack of emotional clarity (Clarity).The DERS has high internal consistency; Cronbach’s α = 0.93 for total DERS & Cronbach’s α>:80 for each factors; test-retest = 0.87 for total DERS & ranging from 0.69 to 0.89 for all factors (9). In an Iranian normal sample, internal consistency of the scale using Cronbach’s α ranged from 0.66 to 0.88 for all factors (11). In relation to validity, studies have suggested sufficient construct and predictive validity for the scale (9). The process of translation and cultural adaptation of the Persian version has been done (11).

Toronto Alexithymia Scale (TAS-20): The twenty-item Toronto Alexithymia Scale (TAS-20) developed by Bagby et al. (1994) is used for measuring the alexithymia construct. It is composed of three dimensions, including difficulty identifying feelings (DIF), difficulty describing feelings (DDF) and externally oriented thinking (EOT) (27). Items are rated on a five-point Likert scale, ranging from 1 (strongly disagree) to 5 (strongly agree). A score more than 60 shows high alexithymia and a score less than 52 shows low alexithymia (28). The internal consistency reliability of the TAS-20 have been reported based on Cronbach-α = 0.79 in a normal sample, and based on test-retest = 0.77 in clinical sample (29). Also, reliability and validity of the scale have been approved in FGID patients and Cronbach’s α = 0.84 has been calculated (30).

Short form of Depression, Anxiety, Stress Scale (DASS): The initial version of the DASS developed by Lovibond & Lovibond in 1995 contains 42 phrases about negative emotional states. The scale measures the intensity of depression, anxiety, stress symptoms, and it can be used to assess treatment progression. Subject rates intensity (frequency) of symptom presented in each phrase which he/she has experienced over the past week on a four-point Likert scale ranges from 0 (Did not apply to me at all) to 3 (Applied to me very much) (31). Short form of the DASS has 21 items and each of its subscales consists of 7 items (32). The internal consistency reliability of the scale was computed in an Iranian sample, and Cronbach's α for depression, anxiety and stress were 0.81, 0.74, 0.78, respectively (33). Also, reliability and validity of the scale have been reviewed and approved in a normal population of Isfahan (34).

*Data Analyses*

To evaluate the construct validity or the factor structure of the scales, the Principal Component Analysis (PCA) method with Varimax Rotation was used to evaluate the internal consistency reliability, Cronbach's Alpha Coefficients were utilized; and to assess concurrent validity, correlation coefficients of the scales with TAS-20 and Short form of the DASS were used. All these calculations were done using statistical- computational software of statistical package for social sciences (SPSS-16).

## Results

*Factor Structure*

The construct validity or the factor structure of the two forms of the DERS (DERS-6 & DERS-5-revised) was performed through principal component analysis and varimax rotation. At First, adequacy of the sample size was confirmed by two tests of sampling adequacy, KMO & Bartlett. The results of KMO test was 0.82 for DERS-6 and 0.84 for DERS-5-revised, which are good (35). Also, Bartlett's test was statistically significant for both the DERS-6 (x = 2/988E2, df = 630, p<0.001) and DERS-5-revised (x = 2/420E2, df = 435, p<0.001). Bartlett's test revealed that correlation matrix between variables in community represents a unit matrix (36).

Results of the factor structure of the two forms of the DERS are represented in Tables 1 and 2. The principal components of orthogonal rotation were analyzed with varimax method. Further, loads greater than 0.30 were considered as significant. However, in case that an item was in more than one factor, based on the greater loading, the researcher classified it in one factor. Finally, 7 factors for DERS-6 and 6 factors for DERS-5-revised were identified in the clinical sample which explained 59.51% and 59.25% of the common variance, respectively. Therefore, the 6 factor DERS complies with Gratz & Roemer (2004) pattern (9), and the 5 factor DERS Complies with Bardeen et al. (2012) pattern (23), but they were not confirmed in an Iranian clinical sample.

**Table 1:** Results of Factor Analysis and Intercolaration of the Items of the DERS-6 in the Clinical Sample

Explained variance%	Factors								Intercolaration of the items	
	Goal	Awareness	Impulse	Non-Accep		Strategy	Clarity	Recognition	Intercolaration of each item with related factor	Intercolaration of each item with total score
	24.982	12.163	5.786	5.092		4.982	3.324	3.182		
Item		Factor Loadings for the 33 Items Included in the Final Factor Analysis								
26	0.693								**0.817	**0.604
18	0.720								**0.830	**0.619
13	0.747								**0.859	**0.690
33	0.726								**0.837	**0.668
15		0.348							**0.379	**0.281
6		0.780							**0.754	**0.333
2		0.818							**0.812	**0.353
10		0.755							**0.744	**0.259
17		0.660							**0.714	**0.215
8		0.640							**0.663	**0.612
32			0.710						**0.353	**0.233
27			0.691						**0.276	**0.546
14			0.844						**0.434	**0.629
19			0.809						**0.440	**0.650
3			0.640						*0.156	**0.660
24			0.478						**0.320	**0.468
25					0.797				**0.792	**0.542
21					0.850				**0.799	**0.526
12					0.645				**0.743	**0.495
11					0.519				**0.735	**0.601
29					0.586				**0.751	**0.538
23					0.504				**0.713	**0.562
16						0.514			**0.781	**0.561
15						0.535			**0.764	**0.568
35						0.622			**0.623	**0.333
28						0.689			**0.698	**0.485
22						0.505			**0.172	**0.361
30							0.364		**0.682	**0.553
5							0.682		**0.742	**0.474
4							0.628		**0.701	**0.302
9							0.462		**0.750	**0.599
7								0.751	**0.986	**0.287
1								0.728	**0.937	**0.254

DERS-6: Difficulties in Emotion Regulation Scale (Scale of 6-factor)

** P<0.01,

* P<0.05

**Table 2:** Results of Factor Analysis and Intercolaration of the Items of the DERS-5-Revised in the Clinical Sample

**Explaind variance%**	**Factors**						**Intercolarationof the items**	
	**Non-Accep**	**Goal**	**Impulse**	**Strategy**	**Clarity**	**Recognition**	**Intercolaration ** **of each item ** **with ** **Related** **factor**	**Intercolaration ** **of each item ** **with total ** **score**
	**28.882**	**8.886**	**6.638**	**5.555**	**4.886**	**4.410**		
**item**	**Factor Loadings for the 29 Items Included in the Final Factor Analysis**							
25	0.783						**0.775	**0.586
21	0.833						**0.775	**0.558
12	0.723						**0.726	**0.530
11	0.577						**0.716	**0.636
29	0.655						**0.761	**0.577
23	0.582						**0.710	**0.599
16	0.383						**0.622	**0.624
26		0.712					**0.797	**0.638
18		0.737					**0.795	**0.656
13		0.726					**0.850	**0.716
33		0.718					**0.803	**0.681
15		0.394					**0.688	**0.634
32			0.721				**0.808	**0.595
27			0.710				**0.777	**0.549
14			0.822				**0.873	**0.632
19			0.780				**0.854	**0.655
3			0.622				**0.762	**0.646
24				0.593			**0.655	**0.404
20				0.503			**0.532	**0.253
35				0.363			**0.571	**0.375
28				0.486			**0.681	**0.532
22				0.723			**0.626	**0.289
36					0.468		**0.609	**0.334
30					0.437		**0.707	**0.598
5					0.659		**0.691	**0.492
4					0.730		**0.638	**0.306
9					0.598		**0.727	**0.605
7						0.866	**0.948	*0.194
1						0.867	**0.937	*0.151

DERS-5 : Difficulties in Emotion Regulation Scale (Scale of 5-factor)

** P<0.01,

* P<0.05

**Table 3:** Descriptive Statistics, Internal Consistency and Correlation Coefficients of Concurrent Validity of the DERS-6 and its Factors

**Variable**	**Descriptive** ** Statistic**		**Reliability**	**Correlation Coefficients of** ** Concurrent Validity**			
	**SD**	**Mean**	**Cronbach’s α**	**Stress**	**Anxiety**	**Depression**	**Alexithymia**
DERS-6	20.142	96.944	0.90	**0.646	**0.606	**0.561	**0.597
Goal	4.008	14.188	0.85	**0.533	**0.511	**0.419	**0.380
Awareness	4.791	15.133	0.77	0.052	0.008	*0.180	**0.292
Impulse	5.825	16.061	0.86	**0.510	**0.493	**0.375	**0.374
Non-Acceptance	6.181	18.933	0.85	**0.480	**0.500	**0.427	**0.396
Strategy	4.458	16.150	0.71	**0.460	**0.456	**0.369	**0.345
Clarity	3.824	11.283	0.69	**0.479	**0.454	**0.391	**0.589
Recognition	2.293	5.044	0.87	*0.177	0.028	*0.186	**0.238

DERS-6: Difficulties in Emotion Regulation Scale (Scale of 6-factor)

** P<0.01,

* P<0.05

**Table 4:** Descriptive statistic, internal consistency & Correlation Coefficients of concurrent validity of The DERS-5-revised and its factors

Variable	Dscriptive Statistic		Reliability	Correlation Coefficients of concurrent validity			
		
	SD	Mean	Cronbach’s α	Stress	Anxiety	Depression	Alexithymia
DERS-5	19.415	87.977	0.90	**0.665	**0.634	**0.550	**0.567
Non-Acceptance	6.948	22.350	0.85	**0.498	**0.519	**0.440	**0.400
Goal	4.748	17.305	0.85	**0.577	**0.545	**0.458	**0.417
Impulse	5.211	13.033	0.87	**0.514	**0.506	**0.358	**0.371
Strategy	3.830	15.850	0.59	**0.412	**0.376	**0.442	**0.320
Clarity	4.482	14.394	0.70	**0.458	**0.445	**0.331	**0.552
Recognition	2.293	5.044	0.87	*0.177	0.028	*0.186	**0.238

DERS-5: Difficulties in Emotion Regulation Scale (Scale of 5-factor)

** P<0.01,

* P<0.05

**Table 5:** Correlations of the Difficulties in Emotion between the Factors Regulation Scale (DERS-6)

Variables	1	2	3	4	5	6
DERS -Non-Accept	-	.559**	.392**	-.155*	.507**	.257**
DERS –Goal		-	.556**	-.031	.543**	.282**
DERS –Impulse			-	.127	.481**	.391**
DERS –Awareness				-	.142-	.314**
DERS –Strategy					-	.288**
DERS –Clarity						-

DERS-6: Difficulties in Emotion Regulation Scale (Scale of 6-factor)

** P<0.01,

* P<0.05

In the study, the factors of DERS-6 were composed of goal, awareness, impulse, non-acceptance, strategy, clarity, and recognition. Items 1 and 7 were loaded onto a separate factor which was titled as “Lack of Emotional Recognition"; Item 15 was transferred to the awareness and item 30 was transferred to the clarity; Item 34 was eliminated because of negative loading and negatively correlating with the total score; and items 31 and 36 were excluded due to singly loading on a factor. On the other hand, 9 factors revealed the DERS-6 that two factors were eliminated due to loading one item. After these changes, the number of the items of this scale was reduced from 36 to 33 items in the clinical sample.

Also, the factors of DERS-5-revised were composed of non-acceptance, goal, impulse, strategy, clarity and recognition. Similar to the DERS-6, items 1 and 7 were loaded onto a separate factor titled “Recognition”, Item 16 to non-acceptance, Item 15 to goal, Items 20 and 24 to strategy and Items 30 and 36 to clarity; Item 31 was excluded due to singly loading on a factor. Therefore, the number of the items of this scale was reduced from 30 to 29 items in the clinical sample.

Intercolaration of each item was computed with the related factor and the total score. As demonstrated in Tables 3 and 4, the results reveal that intercolaration of all items of the DERS-6 and the DERS-5-revised are significant. The findings reveal the desirable construct validity of the two forms of the DERS.

*Concurrent Validity*

In order to assess concurrent validity of the two scales, correlation coefficients between the DERS-6 and the DERS-5-revised and symptom measures (short form of the DASS and the TAS-20) were computed. As demonstrated in Tables 3 and 4, the two forms of the DERS total scale and their factors showed an almost similar pattern of correlations with the symptom measures.

*Reliability*

Cronbach’s α was calculated to determine the internal consistency of the two forms of the DERS total scale and their factors. Results indicated that both the DERS-6 and the DERS-5-revised had good internal consistency (α = 0.90). Also, most of the factors had adequate internal consistency. However, the “strategy factor” in the DERS-5-revised had a stronger internal consistency. Results of the internal consistency and descriptive statistic are presented in Tables 3 and 4.

*Additional Result*

Correlations between the factors of the DERS-6 were computed. As showed in Table 5, most the factors of the DERS-6, except for awareness, were positively correlated. Awareness was negatively correlated with non-acceptance and was positively correlated with clarity.

## Discussion

The purpose of this study was to determine the factor structure and psychometric properties of the two forms of the Persian version of the Difficulties in Emotion Regulation Scale (i.e., DERS-6 & DERS-5-Revised) in a clinical sample. In the factor structure, 7 factors for the DERS-6, and 6 factors for the DERS-5-revised were recognized. The findings did not support the six-factor model developed by Gratz & Roemer (9),but other studies confirmed it (11,17,18,19,20,21), and the revised five-factor model was developed by Bardeen et al. (23) on normal samples; However, the findings are consistent with the results of the above studies based on the multifunctionality of the DERS. 

In explaining these results, it can be stated that in this study, the clarity factor was divided into the two factors of clarity and recognition. This is probably due to the existence of more severe emotional problems in clinical groups, and it suggests further distinctions between the factors involved in emotion regulation. Therefore, forasmuch as emotional problems and difficulties in emotion regulation play an important role in psychosomatic disorders (12, 37), the lack of emotional recognition as a separable factor could cause emotional dysregulation. 

Moreover, concurrent validity of both the DERS-6 & DERS-5-revised was very good. This means that the two forms of the DERS and most of their factors converge with variables related to the extent of emotion regulation. On the other hand, it demonstrates a relatively convergent pattern of correlations with outcomes relevant to the emotion regulation domain (depression, anxiety, stress, alexithymia). The findings are consistent with those of Gratz & Roemer (9), Bardeen et al. (23) and the other studies (11, 18, 19, 20, and 21).

In this study, the factors of the lack of awareness and lack of recognition were demonstrated as weakness factors in relation to emotional problems, because these factors showed only a strong and significant correlation with alexithymia but were not significantly correlated with other emotional symptoms, especially with anxiety which is the underlying emotion of anxiety disorders. This finding confirms the DERS-5-revised model; it can be noted that attention to emotional states does not necessarily represent a normal reaction or regulation of such cases (23). As Tull et al. (2010 & 2007) have mentioned, some forms of emotional awareness may be adaptive (accepting without judgment) and some forms are probably maladaptive (e.g., rumination or negative emotion) (25, 26). Therefore, the awareness factor may measure emotional awareness, but may not be necessarily associated with adaptive emotion regulation (22). And this can be true about the lack of recognition factor.

Finally, the reliability coefficients of the two scale of the DERS-6 and DERS-5-revised and their factors in the clinical sample were desirable or satisfactory; and this finding is consistent with the findings of Gratz & Roemer (9), and Bardeen et al. (23).

**Limitations**

The main limitations of this study were as follows: (a) the clinical group was not compared with a normal sample; (b) the cut off of both forms of the DERS was not determined for the same reason. Due to the lack of the effective role of awareness on emotion regulation, it is recommended that from DERS-5-revised model be used in the clinical groups. Furthermore, since the items 1 and 7 in both scales were loaded on a separate factor (recognition), and this factor had a poor concurrent validity, it is suggested that the recognition factor be eliminated and properties of both DERS be studied in other clinical groups.

## Conclusions

The results of this study are practical and valuable from some aspects: (1) the findings of this study confirmed those of other studies that argued emotion regulation consists of different variables. (2) In clinical assessment, both forms of the DERS can be used as good scales to measure difficulties in emotion regulation. (3) Among the extracted factors, the non-acceptance factor in the DERS-5-revised and the goal factor in the DERS-6 revealed the highest percentage of variance. Therefore, the findings indicated the importance of these factors in emotional regulation as well as the necessity of paying more attention to them in emotion-based therapies. (4) In the studied sample, the number of women was more than men (5 times), so it could be corroborant of higher incidence of emotional problems in women. Hence, the results of this study may apply more to women.
